# 5,7-Dimeth­oxy-2-phenyl-4*H*-chromen-4-one

**DOI:** 10.1107/S1600536809003948

**Published:** 2009-02-11

**Authors:** Angannan Nallasivam, Munirathinam Nethaji, Nagarajan Vembu, Venkatraman Ragunathan, Nagarajan Sulochana

**Affiliations:** a Department of Chemistry, National Institute of Technology, Tiruchirappalli 620 015, India; b Department of Inorganic and Physical Chemistry, Indian Institute of Science, Bangalore 560 012, India; c Department of Chemistry, Urumu Dhanalakshmi College, Tiruchirappalli 620 019, India; d Department of Chemistry, Kandasamy Kandar College, Velur 638 182, India

## Abstract

The asymmetric unit of the title compound, C_17_H_14_O_4_, contains two independent mol­ecules which differ in the relative orientations of the phenyl rings with repect to the essentially planar [maximum deviations of 0.029 (2) and 0.050 (2) Å in the two mol­ecules] chromene fused-ring system, forming dihedral angles of 10.3 (5) and 30.86 (5)° in the two mol­ecules. The crystal structure is stabilized by weak C—H⋯O and C—-H⋯π inter­actions, and π–π stacking inter­actions.

## Related literature

For the biological and pharmacological properties of benzopyrans and their derivatives, see Brooks (1998 ▶); Hatakeyama *et al.* (1988 ▶); Hyana & Saimoto (1987 ▶); Tang *et al.* (2007 ▶). For the importance of 4*H*-chromenes, see Liu *et al.* (2007 ▶); Wang, Fang *et al.* (2003 ▶); Wang, Zhang *et al.* (2003 ▶). For hydrogen bonding, see: Bernstein *et al.* (1995 ▶); Desiraju (1989 ▶); Desiraju & Steiner (1999 ▶); Etter (1990 ▶).
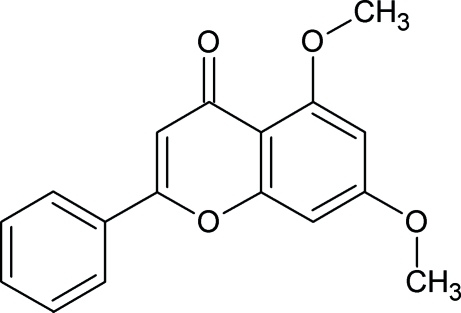


## Experimental

### 

#### Crystal data

C_17_H_14_O_4_
*M* *_r_* = 282.28
Triclinic, 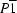

*a* = 7.3938 (17) Å
*b* = 11.430 (3) Å
*c* = 16.547 (4) Å
α = 92.414 (4)°
β = 102.723 (4)°
γ = 91.916 (4)°
*V* = 1361.5 (6) Å^3^
*Z* = 4
Mo *K*α radiation
μ = 0.10 mm^−1^
*T* = 293 (2) K
0.35 × 0.32 × 0.29 mm

#### Data collection

Bruker SMART APEX CCD diffractometer
Absorption correction: multi-scan (*SADABS*; Sheldrick, 1996 ▶) *T*_min_ = 0.967, *T*_max_ = 0.977
15401 measured reflections
6096 independent reflections
3897 reflections with *I* > 2σ(*I*)
*R*_int_ = 0.042

#### Refinement

*R*[*F* ^2^ > 2σ(*F* ^2^)] = 0.050
*wR*(*F* ^2^) = 0.126
*S* = 1.01
6096 reflections
386 parameters
H-atom parameters constrained
Δρ_max_ = 0.17 e Å^−3^
Δρ_min_ = −0.17 e Å^−3^

### 

Data collection: *SMART* (Bruker, 2007 ▶); cell refinement: *SAINT* (Bruker, 2007 ▶); data reduction: *SAINT*; program(s) used to solve structure: *SHELXS97* (Sheldrick, 2008 ▶); program(s) used to refine structure: *SHELXL97* (Sheldrick, 2008 ▶); molecular graphics: *PLATON* (Spek, 2003 ▶); software used to prepare material for publication: *SHELXL97*.

## Supplementary Material

Crystal structure: contains datablocks I, global. DOI: 10.1107/S1600536809003948/lh2766sup1.cif

Structure factors: contains datablocks I. DOI: 10.1107/S1600536809003948/lh2766Isup2.hkl

Additional supplementary materials:  crystallographic information; 3D view; checkCIF report

## Figures and Tables

**Table 1:** Hydrogen-bond geometry (Å, °)

*D*—H⋯*A*	*D*—H	H⋯*A*	*D*⋯*A*	*D*—H⋯*A*
C17*A*—H17*A*⋯O1*A*	0.93	2.38	2.713 (2)	101
C13*B*—H13*E*⋯O11*A*^i^	0.96	2.37	3.169 (2)	141
C19*A*—H19*A*⋯O11*B*^ii^	0.93	2.57	3.256 (2)	131
C19*A*—H19*A*⋯O12*B*^ii^	0.93	2.55	3.442 (2)	161
C15*B*—H15*D*⋯*Cg*5	0.96	2.85	3.786	166

Symmetry codes: (i) 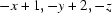; (ii) 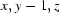. *Cg*5 is the centroids of the C16*A*–C21*A* ring.

**Table 2:** π–π Stacking interactions (Å, °)

*Cg_i_*	*Cg_j_*	*Cg_i_*⋯*Cg_j_*	α	perp
*Cg*1	*Cg*2	3.972 (1)	10.9	3.575
*Cg*1	*Cg*4	3.646 (1)	8.1	3.578
*Cg*1	*Cg*4^i^	3.785 (1)	8.1	3.535
*Cg*2	*Cg*3^i^	3.792 (1)	9.9	3.599
*Cg*2	*Cg*3	3.883 (1)	9.9	3.743
*Cg*3	*Cg*4^i^	3.769 (1)	7.1	3.516

Symmetry code: (i) 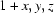. *Cg*1, *Cg*2, *Cg*3, and *Cg*4 are the centroids of the O1*A*/C2*A*–C4*A*/C9*A*/C10*A*, O1*B*/C2*B*–C4*B*/C9*B*/C10*B*, C5*A*–C10*A* and C5*B*–C10*B* rings, respectively. α is the dihedral angle between ring planes and perp is the perpendicular distance between ring planes.

**Fig. 1.**
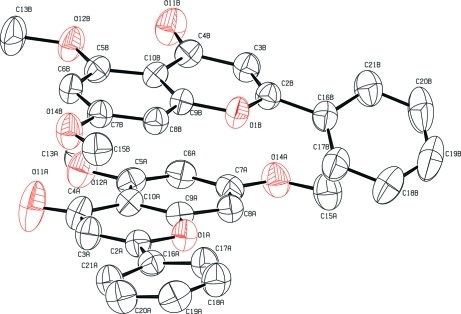
The asymmetric unit of the title compound with displacement ellipsoids shown at the 50% probability level for all non-H atoms. H-atoms are drawn as spheres of arbitrary radius.

**Fig. 2.**
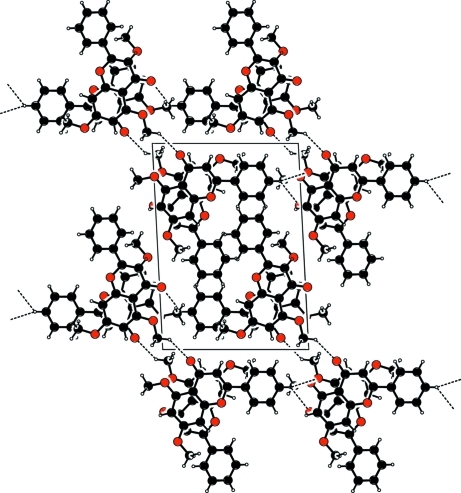
Part of the crystal structure viewed along the a-axis. Dashed lines represent weak C–H···O interactions.

## supplementary crystallographic information

### Comment

Chromenes (benzopyrans) and their derivatives have numerous biological and pharmacological properties (Tang *et al.*, 2007) such as antisterility (Brooks, 1998) and anticancer activity (Hyana & Saimoto, 1987). In addition, polyfunctionalized chromene units are present in numerous natural products (Hatakeyama *et al.*, 1988). 4*H*-chromenes are important synthons for some natural products (Liu *et al.*, 2007). As a part of our structural investigations on 4*H*-chromene derivatives and compounds containing the benzopyran fragment, the single-crystal X-ray diffraction study on the title compound was carried out.

The two molecules (A and B) in the asymmetric unit are shown in Fig. 1. In each molecule, the chromene ring is essentially planar as found in the related chromene derivatives (Wang, Zhang *et al.*, 2003; Wang, Fang *et al.*, 2003). In the title compound, the maximun deviation for the chromene ring in each molecule is 0.029 (2) and 0.050 (2) Å, for atoms C4A and C4B, respectively. The dihedral angle between the chromene ring mean-plane and the phenyl ring is 10.3 (5)° in molecule A and 30.86 (5)° in molecule B. The methoxy substituent at C5 forms dihedral angles of 8.4 (1) and 2.1 (1)° in molecules A and B, respectively. The methoxy substituent at C7 forms dihedral angles of 1.75 (1) & 12.09 (8)° in molecules A and B, respectively.

In the crystal structure (see Fig.2), the intramolecular C17A–H17A···O1A interaction generates a S(5) ring motif (Bernstein *et al.*, 1995; Etter, 1990) and the C19A–H19A···O11B^ii^ and C19A—H19A···O12B^ii^ interactions constitute two bifurcated weak hydrogen bonds bonds that generate a *R*^2^_1_(6) ring motif. In addition, the crystal structure contains significant C—H···π interactions (Table 1) and π..π interactions (Table 2) whose distances agree with those described by Desiraju & Steiner (1999) and Desiraju (1989).

### Experimental

In to the RBF, a suspension of chrysin (1 g, 3.93 mmol) and potassium carbonate (1.62 g, 11.81 mmol) in dimethyl formamide (10 ml) were added. The reaction mixture was heated to 383 K for 2–3 hrs. The reaction mixture was cooled to 313 K and methyl iodide (10 ml, 15.74 mmol) was slowly added with the help of dropping funnel. The reaction mixture was maintained for 8–9 hr at 313 K and monitored by HPLC. After completion of the reaction, the contents were quenched with water and stirred for 30–45 min at 303 K. The crude solid obtained was filtered and washed with plenty of water followed by methanol and dried under vacuum at 343 K. The compound was purified by column chromatography using ethyl acetate: n-hexane (30:70) as diluent. All the fractions were analyzed by HPLC. The highly pure column fractions were mixed and concentrated in a rotary evaporator. The resulting product was recrystallized from dichloromethane: n-hexane mixture (10 ml each). The obtained crystals were washed with n-hexane and dried under vacuum at 348 K. Yield: 70%

### Refinement

All H atoms were observed in a difference Fourier map. However, they were placed in idealized positions with C–H = 0.93 and 0.96 Å for aryl and methyl H, respectively. Their isotropic displacement parameters were tied to common free variables which were refined in subsequent cycles (for the aryl H-atoms the free variable was set to 0.06 which refined to 0.0716 (16) Å^2^ and for the methyl H-atoms the free variable was set to 0.07 and refined to 0.0795 (19) Å^2^).

### Crystal data

**Table:**

C_17_H_14_O_4_	*Z* = 4
*M**_r_* = 282.28	*F*(000) = 592
Triclinic, *P*1	*D*_x_ = 1.377 Mg m^−^^3^
Hall symbol: -P 1	Melting point = 413–415 K
*a* = 7.3938 (17) Å	Mo *K*α radiation, λ = 0.71073 Å
*b* = 11.430 (3) Å	Cell parameters from 547 reflections
*c* = 16.547 (4) Å	θ = 2.7–27.0°
α = 92.414 (4)°	µ = 0.10 mm^−^^1^
β = 102.723 (4)°	*T* = 293 K
γ = 91.916 (4)°	Rectangular, colourless
*V* = 1361.5 (6) Å^3^	0.35 × 0.32 × 0.29 mm

### Data collection

**Table:**

Bruker SMART APEX CCD diffractometer	6096 independent reflections
Radiation source: fine-focus sealed tube	3897 reflections with *I* > 2σ(*I*)
graphite	*R*_int_ = 0.042
Detector resolution: 0.3 pixels mm^-1^	θ_max_ = 28.0°, θ_min_ = 2.1°
ω scans	*h* = −9→9
Absorption correction: multi-scan (*SADABS*; Sheldrick, 1996)	*k* = −14→14
*T*_min_ = 0.967, *T*_max_ = 0.977	*l* = −21→21
15401 measured reflections	

### Refinement

**Table:**

Refinement on *F*^2^	Secondary atom site location: difference Fourier map
Least-squares matrix: full	Hydrogen site location: inferred from neighbouring sites
*R*[*F*^2^ > 2σ(*F*^2^)] = 0.050	H-atom parameters constrained
*wR*(*F*^2^) = 0.126	*w* = 1/[σ^2^(*F*_o_^2^) + (0.0509*P*)^2^ + 0.201*P*] where *P* = (*F*_o_^2^ + 2*F*_c_^2^)/3
*S* = 1.01	(Δ/σ)_max_ < 0.001
6096 reflections	Δρ_max_ = 0.17 e Å^−^^3^
386 parameters	Δρ_min_ = −0.17 e Å^−^^3^
0 restraints	Extinction correction: *SHELXL97* (Sheldrick, 2008), Fc^*^=kFc[1+0.001xFc^2^λ^3^/sin(2θ)]^-1/4^
Primary atom site location: structure-invariant direct methods	Extinction coefficient: 0.051 (3)

### Special details

**Table:**

Geometry. All e.s.d.'s (except the e.s.d. in the dihedral angle between two l.s. planes) are estimated using the full covariance matrix. The cell e.s.d.'s are taken into account individually in the estimation of e.s.d.'s in distances, angles and torsion angles; correlations between e.s.d.'s in cell parameters are only used when they are defined by crystal symmetry. An approximate (isotropic) treatment of cell e.s.d.'s is used for estimating e.s.d.'s involving l.s. planes.
Refinement. Refinement of *F*^2^ against ALL reflections. The weighted *R*-factor *wR* and goodness of fit *S* are based on *F*^2^, conventional *R*-factors *R* are based on *F*, with *F* set to zero for negative *F*^2^. The threshold expression of *F*^2^ > σ(*F*^2^) is used only for calculating *R*-factors(gt) *etc*. and is not relevant to the choice of reflections for refinement. *R*-factors based on *F*^2^ are statistically about twice as large as those based on *F*, and *R*- factors based on ALL data will be even larger.

### Fractional atomic coordinates and isotropic or equivalent isotropic displacement parameters (Å^2^)

**Table:**

	*x*	*y*	*z*	*U*_iso_*/*U*_eq_	
O1A	0.84670 (17)	0.61660 (10)	0.25891 (7)	0.0474 (3)	
C2A	0.7660 (2)	0.57055 (15)	0.18112 (10)	0.0424 (4)	
C3A	0.7470 (3)	0.63612 (16)	0.11527 (11)	0.0511 (5)	
H3A	0.6915	0.6014	0.0635	0.0716 (16)*	
C4A	0.8077 (3)	0.75819 (17)	0.11977 (12)	0.0534 (5)	
C5A	0.9527 (3)	0.92519 (15)	0.22512 (12)	0.0482 (4)	
C6A	1.0295 (3)	0.96129 (16)	0.30639 (12)	0.0506 (5)	
H6A	1.0710	1.0390	0.3193	0.0716 (16)*	
C7A	1.0455 (3)	0.88267 (16)	0.36930 (11)	0.0471 (4)	
C8A	0.9833 (2)	0.76767 (15)	0.35194 (11)	0.0459 (4)	
H8A	0.9931	0.7148	0.3937	0.0716 (16)*	
C9A	0.9053 (2)	0.73324 (14)	0.26986 (11)	0.0416 (4)	
C10A	0.8873 (2)	0.80697 (15)	0.20411 (11)	0.0437 (4)	
O11A	0.7924 (3)	0.81279 (13)	0.05644 (9)	0.0885 (6)	
O12A	0.9347 (2)	0.99695 (11)	0.16087 (8)	0.0626 (4)	
C13A	1.0217 (3)	1.11127 (17)	0.17698 (14)	0.0670 (6)	
H13A	0.9654	1.1550	0.2150	0.0795 (19)*	
H13B	1.0068	1.1507	0.1260	0.0795 (19)*	
H13C	1.1515	1.1051	0.2008	0.0795 (19)*	
O14A	1.1259 (2)	0.92860 (11)	0.44691 (8)	0.0607 (4)	
C15A	1.1399 (3)	0.85301 (18)	0.51405 (12)	0.0651 (6)	
H15A	1.0180	0.8247	0.5172	0.0795 (19)*	
H15B	1.1978	0.8955	0.5650	0.0795 (19)*	
H15C	1.2131	0.7879	0.5052	0.0795 (19)*	
C16A	0.7055 (2)	0.44676 (15)	0.18240 (10)	0.0419 (4)	
C17A	0.7040 (3)	0.39431 (16)	0.25611 (11)	0.0525 (5)	
H17A	0.7461	0.4370	0.3062	0.0716 (16)*	
C18A	0.6406 (3)	0.27909 (17)	0.25620 (13)	0.0596 (5)	
H18A	0.6410	0.2451	0.3063	0.0716 (16)*	
C19A	0.5772 (3)	0.21453 (17)	0.18291 (13)	0.0583 (5)	
H19A	0.5341	0.1372	0.1832	0.0716 (16)*	
C20A	0.5780 (3)	0.26564 (17)	0.10883 (13)	0.0591 (5)	
H20A	0.5342	0.2227	0.0589	0.0716 (16)*	
C21A	0.6430 (3)	0.37968 (16)	0.10844 (11)	0.0521 (5)	
H21A	0.6455	0.4125	0.0582	0.0716 (16)*	
O1B	0.47575 (17)	0.66672 (10)	0.35030 (7)	0.0481 (3)	
C2B	0.5506 (2)	0.75030 (15)	0.41057 (10)	0.0430 (4)	
C3B	0.5514 (3)	0.86351 (16)	0.39514 (11)	0.0482 (4)	
H3B	0.5994	0.9175	0.4389	0.0716 (16)*	
C4B	0.4812 (2)	0.90719 (15)	0.31364 (11)	0.0458 (4)	
C5B	0.3403 (2)	0.83268 (15)	0.16260 (11)	0.0432 (4)	
C6B	0.2695 (3)	0.73982 (16)	0.10767 (11)	0.0474 (4)	
H6B	0.2246	0.7531	0.0519	0.0716 (16)*	
C7B	0.2645 (2)	0.62654 (15)	0.13467 (10)	0.0439 (4)	
C8B	0.3323 (2)	0.60447 (15)	0.21647 (10)	0.0425 (4)	
H8B	0.3293	0.5289	0.2350	0.0716 (16)*	
C9B	0.4054 (2)	0.69884 (15)	0.27057 (10)	0.0397 (4)	
C10B	0.4104 (2)	0.81474 (14)	0.24840 (10)	0.0407 (4)	
O11B	0.4835 (2)	1.01305 (11)	0.30281 (8)	0.0661 (4)	
O12B	0.34727 (19)	0.94462 (10)	0.13977 (8)	0.0578 (4)	
C13B	0.2722 (4)	0.96692 (18)	0.05480 (12)	0.0726 (7)	
H13D	0.3437	0.9288	0.0204	0.0795 (19)*	
H13E	0.2766	1.0498	0.0478	0.0795 (19)*	
H13F	0.1457	0.9371	0.0392	0.0795 (19)*	
O14B	0.19111 (19)	0.54206 (11)	0.07452 (7)	0.0582 (4)	
C15B	0.1540 (3)	0.42788 (16)	0.09996 (12)	0.0571 (5)	
H15D	0.2679	0.3959	0.1280	0.0795 (19)*	
H15E	0.0984	0.3781	0.0521	0.0795 (19)*	
H15F	0.0705	0.4326	0.1369	0.0795 (19)*	
C16B	0.6345 (2)	0.69686 (16)	0.48880 (11)	0.0477 (4)	
C17B	0.7041 (3)	0.5870 (2)	0.48775 (13)	0.0708 (6)	
H17B	0.6916	0.5444	0.4375	0.0716 (16)*	
C18B	0.7922 (4)	0.5393 (2)	0.56032 (16)	0.0905 (8)	
H18B	0.8401	0.4653	0.5584	0.0716 (16)*	
C19B	0.8101 (4)	0.5986 (3)	0.63429 (16)	0.0886 (9)	
H19B	0.8713	0.5662	0.6829	0.0716 (16)*	
C20B	0.7371 (4)	0.7066 (3)	0.63683 (13)	0.0845 (8)	
H20B	0.7460	0.7467	0.6877	0.0716 (16)*	
C21B	0.6504 (3)	0.7572 (2)	0.56492 (12)	0.0640 (6)	
H21B	0.6029	0.8313	0.5674	0.0716 (16)*	

### Atomic displacement parameters (Å^2^)

**Table:**

	*U*^11^	*U*^22^	*U*^33^	*U*^12^	*U*^13^	*U*^23^
O1A	0.0561 (8)	0.0411 (7)	0.0418 (7)	−0.0064 (6)	0.0050 (6)	0.0036 (5)
C2A	0.0397 (10)	0.0445 (10)	0.0415 (9)	−0.0022 (8)	0.0068 (8)	0.0014 (8)
C3A	0.0570 (12)	0.0483 (11)	0.0431 (10)	−0.0088 (9)	0.0020 (9)	0.0040 (8)
C4A	0.0564 (12)	0.0493 (11)	0.0490 (11)	−0.0052 (9)	−0.0011 (9)	0.0123 (9)
C5A	0.0462 (11)	0.0412 (10)	0.0572 (11)	0.0008 (8)	0.0108 (9)	0.0066 (9)
C6A	0.0551 (12)	0.0382 (10)	0.0579 (12)	−0.0019 (8)	0.0131 (9)	−0.0017 (9)
C7A	0.0470 (11)	0.0482 (11)	0.0463 (10)	0.0001 (8)	0.0125 (8)	−0.0051 (8)
C8A	0.0487 (11)	0.0457 (11)	0.0431 (10)	0.0013 (8)	0.0098 (8)	0.0023 (8)
C9A	0.0395 (10)	0.0369 (10)	0.0479 (10)	−0.0021 (7)	0.0094 (8)	0.0004 (8)
C10A	0.0410 (10)	0.0407 (10)	0.0475 (10)	−0.0014 (8)	0.0056 (8)	0.0041 (8)
O11A	0.1298 (15)	0.0642 (10)	0.0536 (9)	−0.0281 (9)	−0.0169 (9)	0.0215 (8)
O12A	0.0768 (10)	0.0429 (8)	0.0616 (8)	−0.0094 (7)	0.0014 (7)	0.0124 (6)
C13A	0.0828 (16)	0.0411 (11)	0.0732 (14)	−0.0029 (11)	0.0086 (12)	0.0073 (10)
O14A	0.0793 (10)	0.0523 (8)	0.0473 (8)	−0.0068 (7)	0.0104 (7)	−0.0076 (6)
C15A	0.0802 (16)	0.0634 (14)	0.0472 (11)	−0.0097 (11)	0.0077 (10)	−0.0046 (10)
C16A	0.0382 (10)	0.0424 (10)	0.0455 (10)	0.0002 (8)	0.0103 (8)	0.0033 (8)
C17A	0.0615 (13)	0.0478 (11)	0.0468 (10)	−0.0043 (9)	0.0101 (9)	0.0012 (9)
C18A	0.0715 (14)	0.0504 (12)	0.0575 (12)	−0.0033 (10)	0.0150 (11)	0.0125 (10)
C19A	0.0613 (13)	0.0415 (11)	0.0712 (14)	−0.0060 (9)	0.0139 (11)	0.0045 (10)
C20A	0.0670 (14)	0.0486 (12)	0.0583 (12)	−0.0090 (10)	0.0103 (10)	−0.0081 (10)
C21A	0.0586 (12)	0.0505 (11)	0.0458 (10)	−0.0063 (9)	0.0098 (9)	0.0015 (9)
O1B	0.0634 (8)	0.0400 (7)	0.0363 (6)	0.0004 (6)	0.0005 (6)	0.0056 (5)
C2B	0.0442 (10)	0.0437 (10)	0.0389 (9)	0.0000 (8)	0.0052 (8)	−0.0002 (8)
C3B	0.0537 (12)	0.0429 (11)	0.0447 (10)	−0.0017 (8)	0.0052 (9)	−0.0014 (8)
C4B	0.0467 (11)	0.0385 (10)	0.0503 (10)	−0.0006 (8)	0.0070 (8)	0.0032 (8)
C5B	0.0458 (10)	0.0379 (10)	0.0458 (10)	0.0026 (8)	0.0086 (8)	0.0094 (8)
C6B	0.0551 (11)	0.0485 (11)	0.0368 (9)	0.0023 (9)	0.0054 (8)	0.0084 (8)
C7B	0.0466 (11)	0.0438 (10)	0.0400 (9)	0.0000 (8)	0.0070 (8)	0.0013 (8)
C8B	0.0485 (11)	0.0359 (9)	0.0427 (10)	0.0003 (8)	0.0091 (8)	0.0054 (7)
C9B	0.0419 (10)	0.0410 (10)	0.0357 (9)	0.0024 (7)	0.0066 (7)	0.0059 (7)
C10B	0.0402 (10)	0.0387 (9)	0.0419 (9)	0.0030 (7)	0.0057 (8)	0.0055 (7)
O11B	0.0908 (11)	0.0367 (8)	0.0641 (9)	−0.0052 (7)	0.0034 (8)	0.0062 (6)
O12B	0.0784 (10)	0.0411 (7)	0.0495 (7)	0.0003 (6)	0.0023 (7)	0.0152 (6)
C13B	0.112 (2)	0.0524 (13)	0.0505 (12)	0.0059 (12)	0.0084 (12)	0.0219 (10)
O14B	0.0795 (10)	0.0478 (8)	0.0414 (7)	−0.0099 (7)	0.0034 (6)	−0.0021 (6)
C15B	0.0674 (14)	0.0449 (11)	0.0562 (12)	−0.0079 (9)	0.0101 (10)	−0.0039 (9)
C16B	0.0450 (11)	0.0562 (12)	0.0401 (10)	−0.0037 (9)	0.0058 (8)	0.0066 (8)
C17B	0.0904 (17)	0.0693 (15)	0.0514 (12)	0.0209 (12)	0.0086 (11)	0.0136 (11)
C18B	0.104 (2)	0.0924 (19)	0.0740 (17)	0.0314 (16)	0.0065 (15)	0.0352 (15)
C19B	0.0694 (17)	0.131 (3)	0.0593 (15)	−0.0098 (16)	−0.0052 (12)	0.0459 (17)
C20B	0.0812 (18)	0.126 (2)	0.0370 (11)	−0.0270 (17)	−0.0028 (11)	0.0029 (13)
C21B	0.0662 (14)	0.0742 (15)	0.0465 (11)	−0.0095 (11)	0.0047 (10)	−0.0034 (10)

### Geometric parameters (Å, °)

**Table:**

O1A—C2A	1.369 (2)	O1B—C2B	1.362 (2)
O1A—C9A	1.380 (2)	O1B—C9B	1.3773 (19)
C2A—C3A	1.333 (2)	C2B—C3B	1.329 (2)
C2A—C16A	1.472 (2)	C2B—C16B	1.474 (2)
C3A—C4A	1.445 (3)	C3B—C4B	1.450 (2)
C3A—H3A	0.9300	C3B—H3B	0.9300
C4A—O11A	1.228 (2)	C4B—O11B	1.231 (2)
C4A—C10A	1.468 (3)	C4B—C10B	1.474 (2)
C5A—O12A	1.356 (2)	C5B—O12B	1.3517 (19)
C5A—C6A	1.379 (3)	C5B—C6B	1.378 (2)
C5A—C10A	1.422 (2)	C5B—C10B	1.426 (2)
C6A—C7A	1.392 (2)	C6B—C7B	1.389 (2)
C6A—H6A	0.9300	C6B—H6B	0.9300
C7A—O14A	1.365 (2)	C7B—O14B	1.365 (2)
C7A—C8A	1.375 (2)	C7B—C8B	1.373 (2)
C8A—C9A	1.388 (2)	C8B—C9B	1.387 (2)
C8A—H8A	0.9300	C8B—H8B	0.9300
C9A—C10A	1.390 (2)	C9B—C10B	1.391 (2)
O12A—C13A	1.425 (2)	O12B—C13B	1.430 (2)
C13A—H13A	0.9600	C13B—H13D	0.9600
C13A—H13B	0.9600	C13B—H13E	0.9600
C13A—H13C	0.9600	C13B—H13F	0.9600
O14A—C15A	1.424 (2)	O14B—C15B	1.425 (2)
C15A—H15A	0.9600	C15B—H15D	0.9600
C15A—H15B	0.9600	C15B—H15E	0.9600
C15A—H15C	0.9600	C15B—H15F	0.9600
C16A—C17A	1.384 (2)	C16B—C17B	1.374 (3)
C16A—C21A	1.395 (2)	C16B—C21B	1.390 (3)
C17A—C18A	1.383 (3)	C17B—C18B	1.378 (3)
C17A—H17A	0.9300	C17B—H17B	0.9300
C18A—C19A	1.373 (3)	C18B—C19B	1.352 (4)
C18A—H18A	0.9300	C18B—H18B	0.9300
C19A—C20A	1.381 (3)	C19B—C20B	1.366 (4)
C19A—H19A	0.9300	C19B—H19B	0.9300
C20A—C21A	1.375 (3)	C20B—C21B	1.381 (3)
C20A—H20A	0.9300	C20B—H20B	0.9300
C21A—H21A	0.9300	C21B—H21B	0.9300
			
C2A—O1A—C9A	119.71 (13)	C2B—O1B—C9B	119.73 (13)
C3A—C2A—O1A	120.94 (16)	C3B—C2B—O1B	121.51 (15)
C3A—C2A—C16A	127.26 (16)	C3B—C2B—C16B	127.33 (16)
O1A—C2A—C16A	111.79 (14)	O1B—C2B—C16B	111.08 (15)
C2A—C3A—C4A	123.68 (17)	C2B—C3B—C4B	123.40 (17)
C2A—C3A—H3A	118.2	C2B—C3B—H3B	118.3
C4A—C3A—H3A	118.2	C4B—C3B—H3B	118.3
O11A—C4A—C3A	120.59 (18)	O11B—C4B—C3B	120.83 (16)
O11A—C4A—C10A	124.93 (17)	O11B—C4B—C10B	125.04 (16)
C3A—C4A—C10A	114.47 (16)	C3B—C4B—C10B	114.12 (15)
O12A—C5A—C6A	123.64 (16)	O12B—C5B—C6B	123.15 (15)
O12A—C5A—C10A	115.79 (16)	O12B—C5B—C10B	116.05 (15)
C6A—C5A—C10A	120.57 (17)	C6B—C5B—C10B	120.80 (15)
C5A—C6A—C7A	120.67 (17)	C5B—C6B—C7B	120.73 (16)
C5A—C6A—H6A	119.7	C5B—C6B—H6B	119.6
C7A—C6A—H6A	119.7	C7B—C6B—H6B	119.6
O14A—C7A—C8A	124.12 (17)	O14B—C7B—C8B	123.99 (16)
O14A—C7A—C6A	115.13 (16)	O14B—C7B—C6B	115.29 (15)
C8A—C7A—C6A	120.76 (17)	C8B—C7B—C6B	120.71 (16)
C7A—C8A—C9A	117.70 (16)	C7B—C8B—C9B	117.70 (16)
C7A—C8A—H8A	121.2	C7B—C8B—H8B	121.1
C9A—C8A—H8A	121.2	C9B—C8B—H8B	121.1
O1A—C9A—C8A	113.28 (15)	O1B—C9B—C8B	113.06 (14)
O1A—C9A—C10A	122.35 (15)	O1B—C9B—C10B	122.23 (15)
C8A—C9A—C10A	124.36 (16)	C8B—C9B—C10B	124.70 (15)
C9A—C10A—C5A	115.92 (16)	C9B—C10B—C5B	115.31 (15)
C9A—C10A—C4A	118.78 (16)	C9B—C10B—C4B	118.81 (15)
C5A—C10A—C4A	125.28 (16)	C5B—C10B—C4B	125.86 (15)
C5A—O12A—C13A	117.71 (15)	C5B—O12B—C13B	117.79 (14)
O12A—C13A—H13A	109.5	O12B—C13B—H13D	109.5
O12A—C13A—H13B	109.5	O12B—C13B—H13E	109.5
H13A—C13A—H13B	109.5	H13D—C13B—H13E	109.5
O12A—C13A—H13C	109.5	O12B—C13B—H13F	109.5
H13A—C13A—H13C	109.5	H13D—C13B—H13F	109.5
H13B—C13A—H13C	109.5	H13E—C13B—H13F	109.5
C7A—O14A—C15A	117.48 (15)	C7B—O14B—C15B	117.91 (13)
O14A—C15A—H15A	109.5	O14B—C15B—H15D	109.5
O14A—C15A—H15B	109.5	O14B—C15B—H15E	109.5
H15A—C15A—H15B	109.5	H15D—C15B—H15E	109.5
O14A—C15A—H15C	109.5	O14B—C15B—H15F	109.5
H15A—C15A—H15C	109.5	H15D—C15B—H15F	109.5
H15B—C15A—H15C	109.5	H15E—C15B—H15F	109.5
C17A—C16A—C21A	118.04 (16)	C17B—C16B—C21B	118.44 (18)
C17A—C16A—C2A	121.46 (16)	C17B—C16B—C2B	120.39 (17)
C21A—C16A—C2A	120.47 (15)	C21B—C16B—C2B	121.14 (18)
C18A—C17A—C16A	120.81 (18)	C16B—C17B—C18B	120.7 (2)
C18A—C17A—H17A	119.6	C16B—C17B—H17B	119.7
C16A—C17A—H17A	119.6	C18B—C17B—H17B	119.7
C19A—C18A—C17A	120.55 (18)	C19B—C18B—C17B	120.9 (3)
C19A—C18A—H18A	119.7	C19B—C18B—H18B	119.5
C17A—C18A—H18A	119.7	C17B—C18B—H18B	119.5
C18A—C19A—C20A	119.32 (18)	C18B—C19B—C20B	119.3 (2)
C18A—C19A—H19A	120.3	C18B—C19B—H19B	120.4
C20A—C19A—H19A	120.3	C20B—C19B—H19B	120.4
C21A—C20A—C19A	120.36 (18)	C19B—C20B—C21B	121.0 (2)
C21A—C20A—H20A	119.8	C19B—C20B—H20B	119.5
C19A—C20A—H20A	119.8	C21B—C20B—H20B	119.5
C20A—C21A—C16A	120.90 (17)	C20B—C21B—C16B	119.7 (2)
C20A—C21A—H21A	119.5	C20B—C21B—H21B	120.2
C16A—C21A—H21A	119.5	C16B—C21B—H21B	120.2
			
C9A—O1A—C2A—C3A	1.6 (2)	C9B—O1B—C2B—C3B	2.6 (2)
C9A—O1A—C2A—C16A	−177.46 (14)	C9B—O1B—C2B—C16B	−174.21 (14)
O1A—C2A—C3A—C4A	−0.1 (3)	O1B—C2B—C3B—C4B	−2.6 (3)
C16A—C2A—C3A—C4A	178.82 (18)	C16B—C2B—C3B—C4B	173.59 (17)
C2A—C3A—C4A—O11A	177.3 (2)	C2B—C3B—C4B—O11B	179.30 (18)
C2A—C3A—C4A—C10A	−2.1 (3)	C2B—C3B—C4B—C10B	−0.8 (3)
O12A—C5A—C6A—C7A	−179.36 (17)	O12B—C5B—C6B—C7B	−179.50 (16)
C10A—C5A—C6A—C7A	0.4 (3)	C10B—C5B—C6B—C7B	0.1 (3)
C5A—C6A—C7A—O14A	179.18 (16)	C5B—C6B—C7B—O14B	179.95 (16)
C5A—C6A—C7A—C8A	−0.7 (3)	C5B—C6B—C7B—C8B	−0.9 (3)
O14A—C7A—C8A—C9A	−179.62 (16)	O14B—C7B—C8B—C9B	179.06 (16)
C6A—C7A—C8A—C9A	0.3 (3)	C6B—C7B—C8B—C9B	0.0 (3)
C2A—O1A—C9A—C8A	179.66 (14)	C2B—O1B—C9B—C8B	−179.34 (15)
C2A—O1A—C9A—C10A	−0.7 (2)	C2B—O1B—C9B—C10B	1.1 (2)
C7A—C8A—C9A—O1A	−179.81 (15)	C7B—C8B—C9B—O1B	−177.72 (15)
C7A—C8A—C9A—C10A	0.6 (3)	C7B—C8B—C9B—C10B	1.8 (3)
O1A—C9A—C10A—C5A	179.54 (15)	O1B—C9B—C10B—C5B	176.98 (15)
C8A—C9A—C10A—C5A	−0.9 (3)	C8B—C9B—C10B—C5B	−2.5 (3)
O1A—C9A—C10A—C4A	−1.6 (3)	O1B—C9B—C10B—C4B	−4.5 (2)
C8A—C9A—C10A—C4A	177.98 (16)	C8B—C9B—C10B—C4B	176.01 (16)
O12A—C5A—C10A—C9A	−179.87 (15)	O12B—C5B—C10B—C9B	−178.86 (15)
C6A—C5A—C10A—C9A	0.4 (3)	C6B—C5B—C10B—C9B	1.5 (2)
O12A—C5A—C10A—C4A	1.4 (3)	O12B—C5B—C10B—C4B	2.7 (3)
C6A—C5A—C10A—C4A	−178.39 (17)	C6B—C5B—C10B—C4B	−176.92 (17)
O11A—C4A—C10A—C9A	−176.5 (2)	O11B—C4B—C10B—C9B	−175.93 (17)
C3A—C4A—C10A—C9A	2.9 (3)	C3B—C4B—C10B—C9B	4.1 (2)
O11A—C4A—C10A—C5A	2.2 (3)	O11B—C4B—C10B—C5B	2.5 (3)
C3A—C4A—C10A—C5A	−178.40 (17)	C3B—C4B—C10B—C5B	−177.47 (16)
C6A—C5A—O12A—C13A	8.5 (3)	C6B—C5B—O12B—C13B	1.8 (3)
C10A—C5A—O12A—C13A	−171.26 (17)	C10B—C5B—O12B—C13B	−177.77 (17)
C8A—C7A—O14A—C15A	−2.3 (3)	C8B—C7B—O14B—C15B	11.0 (3)
C6A—C7A—O14A—C15A	177.86 (17)	C6B—C7B—O14B—C15B	−169.90 (16)
C3A—C2A—C16A—C17A	−168.56 (19)	C3B—C2B—C16B—C17B	−148.0 (2)
O1A—C2A—C16A—C17A	10.4 (2)	O1B—C2B—C16B—C17B	28.5 (3)
C3A—C2A—C16A—C21A	9.7 (3)	C3B—C2B—C16B—C21B	30.0 (3)
O1A—C2A—C16A—C21A	−171.29 (15)	O1B—C2B—C16B—C21B	−153.46 (17)
C21A—C16A—C17A—C18A	−0.6 (3)	C21B—C16B—C17B—C18B	−1.8 (3)
C2A—C16A—C17A—C18A	177.72 (17)	C2B—C16B—C17B—C18B	176.3 (2)
C16A—C17A—C18A—C19A	−0.3 (3)	C16B—C17B—C18B—C19B	0.9 (4)
C17A—C18A—C19A—C20A	0.3 (3)	C17B—C18B—C19B—C20B	0.9 (4)
C18A—C19A—C20A—C21A	0.5 (3)	C18B—C19B—C20B—C21B	−1.8 (4)
C19A—C20A—C21A—C16A	−1.5 (3)	C19B—C20B—C21B—C16B	0.9 (4)
C17A—C16A—C21A—C20A	1.5 (3)	C17B—C16B—C21B—C20B	0.9 (3)
C2A—C16A—C21A—C20A	−176.89 (17)	C2B—C16B—C21B—C20B	−177.16 (18)

### Hydrogen-bond geometry (Å, °)

**Table:**

*D*—H···*A*	*D*—H	H···*A*	*D*···*A*	*D*—H···*A*
C17A—H17A···O1A	0.93	2.38	2.713 (2)	101
C13B—H13E···O11A^i^	0.96	2.37	3.169 (2)	141
C19A—H19A···O11B^ii^	0.93	2.57	3.256 (2)	131
C19A—H19A···O12B^ii^	0.93	2.55	3.442 (2)	161
C13A—H13A···Cg5^iii^	0.96	3.14	3.838	131
C15A—H15A···Cg6	0.96	3.16	4.059	156
C15B—H15D···Cg5	0.96	2.85	3.786	166

Symmetry codes: (i) −*x*+1, −*y*+2, −*z*; (ii) *x*, *y*−1, *z*; (iii) *x*, *y*+1, *z*.

### Table 2 π–π Stacking interactions (Å, °)

**Table:**

		Cg···Cg	α	perp
Cg1	Cg2	3.972 (1)	10.88	3.575
Cg1	Cg4	3.646 (1)	8.06	3.578
Cg1	Cg4^i^	3.785 (1)	8.06	3.535
Cg2	Cg3^i^	3.792 (1)	9.89	3.599
Cg2	Cg3	3.883 (1)	9.89	3.743
Cg3	Cg4^i^	3.769 (1)	7.07	3.516

Symmetry code: (i) 1+x, y, z.Cg1, Cg2, Cg3, and Cg4 are the centroids of the O1A/C2A–C4A/C9A/C10A, O1B/C2B–C4B/C9B/C10B, C5A–C10A and C5B–C10B rings, respectively. α is the dihedral angle between ring planes and perp is the perpendicular distance between ring planes.
